# Bilateral Ovarian Krukenberg Tumor in a Full-Term Pregnancy

**DOI:** 10.5402/2011/620380

**Published:** 2010-10-28

**Authors:** Omar Felipe Dueñas-García, Maricela Diaz-Sotomayor, Charu Chanana

**Affiliations:** ^1^Department of Obstetrics and Gynecology, Bronx Lebanon Hospital Center, 1650 Grand Concourse, Bronx, NY, 10457, USA; ^2^Baylor Collage of Medicine, Children's Nutrition Research Center, Houston, TX 77030 Texas, USA

## Abstract

*Objective*. Krukenberg tumors in pregnancy are very rare and their management can present a dilemma for the obstetrician gynecologist. *Case Report*. We present the case of a G3P2002 who presented to us and the 38 weeks gestation with bilateral massive Krukenberg tumors. Despite at surgery and chemotherapy she died 3 months postpartum. *Conclusion*. Early detection followed by surgery and chemotherapy could possibly result in a favorable outcome with such patients.

## 1. Introduction

The presence of adnexal masses during pregnancy ranges from 1 to 81 to 1 in 2,500 pregnancies, but only 3% of these masses are malignant. Krukenberg tumor is an ovarian metastasis of a gastric tumor and accounts for 1%—2% of all ovarian tumors [1]. These tumors during pregnancy are even rarer, as the incidence of gastric cancer in women of reproductive age group is only 0.4%-0.5% [2]. Thus they are a diagnostic and treatment challenge to the physician. We present a case report of a woman 38 weeks pregnant with massive bilateral Krukenberg tumors, which had an unfortunate outcome despite of treatment.

## 2. Case Report

A 30-year-old woman, gravida 3 para 2, from a very remote community was referred at 38 weeks to our tertiary care center due to the presence a bilateral ovarian masses and shortness of breath. The patient complained of history of 1 year before the pregnancy with mild bloating and early satiety that was attributed to gastritis and gastroesophageal reflux, but despite the symptoms she never saught for medical attention. A pelvic sonogram suggested that the ovarian tumors were malignant. Decision to proceed with exploratory laparotomy and cesarean section was taken. Circulating levels were as follows: testosterone (0.93 *μ*g/L, normal range: <0.62 *μ*g/L), progesterone (22.29 nmol/L, normal range: 0.64–2.58 nmol/L), and 17-OH-progesterone (4.96 *μ*g/L, normal range: 0.40–1.02 *μ*g/L), and the tumor markers levels were 43.4 U/mL (CA 19–9), 1.7 ng/mL (carcinoembrionic antigen) 12.8 U/mL (CA72–4) and 33.9 U/mL (CA 125), respectively. 

**Figure 1:**
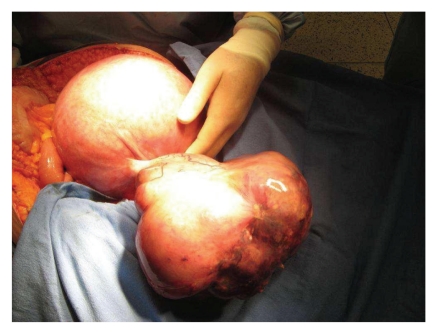
Presence of a bilateral ovarian tumor and a full-term gravid uterus.

Intraoperatively she was found to have two bilateral adnexal masses measuring 15 cm in its greatest diameter for the right ovary and 22 cm for the left (Figure 1) and also a 10 cm tumor in the major gastric curvature that resembled linitis plastica. She underwent an elective cesarean delivery and debulking surgery including bilateral oophorectomy omentectomy, and gastric tumor biopsy. Histopathology of the gastric biopsy was consistent with the presence of poorly differentiated tumor cells corroborating the diagnosis of linitis plastica. Since debulking does not improve the prognosis we decided against debulking. 

In the postoperative period the patient was started on chemotherapy with 6 cycles of chemotherapy using 5-fluorouracil (2000 mg/m^2^) and oxaliplatin (50 mg/m^2^) in a weekly schedule. Despite all our efforts the patient succumbed to disease in 3 months.

## 3. Discussion

Krukenberg tumor refers to gastrointestinal cancer metastatic to the ovaries, accounting for 1%-2% of all ovarian tumors. 

The eponym of this condition was given due by Krukenberg initially described in 1896 and the criteria given were (1) the presence of a tumor in the ovary, (2) evidence of intracellular mucin secretion by the formation of signet cells, and (3) diffuse infiltration of stroma giving a sarcoma-like appearance [2]. 

A search in English, Spanish, and French literature revealed four reports of bilateral ovarian Krukenberg tumors, with our case being the fifth in the literature. 

The cornerstone of the management of these tumors is the diagnosis of the gastrointestinal primary tumor, and also the prognosis worsens when the primary tumor is identified after the metastasis to the ovary is discovered [3]. 

Due to the rare nature of these tumors, there is no current standardization for the diagnosis and the treatment. 

Also due to the rarity of the condition it is not appropriate to comment on the effect of tumor on pregnancy and vice versa. There are only 3 cases of pregnancy-related Krukenberg the tumors in the literature; of these also 2 were detected postpartum, and this is the only tumor that is not associated to virilization. Some authors are of the opinion that there might be an increased risk of acute abdomen secondary to tumor torsion or rupture [4]. 

The role of debulking surgery and chemotherapy with platinum-based chemotherapy can be reasonable, and even relatively safe to be administered during pregnancy. But despite the interventions, usually the discovery of the presence of masses of the size that our patient exhibited the prognosis is poor as it generally represents an advanced stage disease, However, possible early detection with debulking surgery, possible hysterectomy with/without delivery, and platinum-based chemotherapy may improve the survival of these patients [5, 6].
